# Polydatin Inhibits Adipose Tissue Inflammation and Ameliorates Lipid Metabolism in High-Fat-Fed Mice

**DOI:** 10.1155/2019/7196535

**Published:** 2019-11-15

**Authors:** Li Zheng, Jiayuan Wu, Juanfen Mo, Li Guo, Xiaoyan Wu, Yi Bao

**Affiliations:** ^1^The Key Laboratory, The Second Affiliated Hospital of Jiaxing University, Jiaxing, Zhejiang 314000, China; ^2^Clinical Laboratory, The Second Affiliated Hospital of Jiaxing University, Jiaxing, Zhejiang 314000, China

## Abstract

Polydatin (PD), an active component of Chinese herbs, is reported to have many biological functions, such as cardioprotective actions, anti-inflammatory activities, and antitumor effects. In this study, we investigated the effects of PD on body weight control, glucose and lipid metabolic regulation, and anti-inflammation in a high-fat-diet- (HFD-) induced obese mice model. After treatment of PD (100 mg/kg/d for 4 weeks), HFD mice reduced body weight, retroperitoneal fat mass, and adipose cell sizes; significantly lowered serum total cholesterol triglyceride (TG) and low-density lipoprotein (LDL) levels; and increased high-density lipoprotein (HDL) levels compared with the HFD control mice. Further studies showed that PD downregulated the mRNA and protein expressions of peroxisome proliferator-activated receptor gamma (PPAR*γ*), a transcription factor involving in the regulation of adipocyte differentiation, in the retroperitoneal fat of HFD mice. Additionally, PD significantly upregulated the mRNA and protein expressions of leptin, an adipocyte-derived anorexic hormone that regulates food intake and energy expenditure, in the adipose tissues of HFD mice. Moreover, PD reduced the expression levels of monocyte chemoattractant protein-1 (MCP-1) and tumor necrosis factor-alpha (TNF-*α*) in the retroperitoneal and epididymal tissues of HFD mice, suggesting that PD prevented adipose tissue inflammation. In conclusion, PD may serve as a pharmaceutic candidate for obesity-related lipid metabolism, anti-inflammation, and body weight loss.

## 1. Introduction

Obesity is defined as an abnormal or excessive accumulation of fat that remains a global public health problem. Obesity is an important risk factor for metabolic syndromes such as insulin resistance, type 2 diabetes, cardiovascular diseases, nonalcoholic fatty liver diseases, hypertension, and hyperlipidemia [1–3]. Adipose tissue, which is mainly composed of adipocytes, is crucial for maintaining metabolic and energy homeostasis [4]. Adipocyte differentiation is critical for fat accumulation and energy and endocrine homeostasis and is a process that requires the strict control of several related transcription factors [4, 5]. Peroxisome proliferator-activated receptor-*γ* (PPAR*γ*) is a member of the nuclear receptor superfamily of ligand-activated transcription factors and has been verified to be a master regulator of adipocyte differentiation [4–6]. CCAAT/enhancer-binding protein (C/EBP*α*), which functions as a principal player in adipogenesis [4, 5], is incapable of initiating the adipogenic process in the absence of PPAR*γ* [7]. Thus, PPAR*γ* and C/EBP*α* participate in a single pathway of adipocyte differentiation, in which PPAR*γ* is the dominant regulation factor.

Obesity is characterized by adipose tissue dysfunction resulting in increased adipose tissue inflammation, which can cause chronic low-grade systemic inflammation [8, 9]. Obesity is associated with a higher rate for noncommunicable diseases, including type 2 diabetes, cardiovascular diseases, musculoskeletal disorders, and some cancers [2, 9, 10]. White adipose tissue (WAT) is an endocrine organ and its importance for whole-body metabolism has been well-recognized [11]. Adipokines are a wide variety of cytokines secreted by adipose tissues [12], which participate in many pathophysiological processes including the regulation of appetite and satiety, immunity, inflammation adipogenesis, insulin sensitivity, and others [12–14]. More than 600 adipokines have been identified, some of which play a role in chronic inflammation (e.g., MCP-1, TNF-*α*, IL-1*β*, IL-6, IL-8, IL-10) [13]. Dysregulation of adipokine secretion in obesity leads to increased proinflammatory activities locally and systemically [15, 16].

Polydatin (PD, 3,4′,5-trihydroxystilbene-3-*β*-D-glucoside) is a monocrystalline compound firstly isolated from the dried root and rhizome of *Polygonum cuspidatum* Sieb. (Polygonaceae), a widely used traditional Chinese herb [17, 18]. PD is identified as the most abundant precursor of resveratrol in nature [19]. Previous studies have demonstrated that PD has many biological functions, such as cardioprotective actions, anti-inflammatory activities, and antitumor effects [17]. Additionally, PD presents anti-inflammatory effects in the mature adipocyte cells, which might mediate through suppressing MCP-1 and TNF-*α* expression [20]. However, whether and how PD influences glucose and lipid metabolism and inflammation state have not been fully understood in obesity. In this study, we investigated the effects of PD on body weight control, anti-inflammation, and other metabolic parameters in a HFD-induced mice model. Our work showed that PD treatment ameliorated dysfunction of lipid metabolism and inhibited inflammation state in the adipose tissues of obese mice.

## 2. Materials and Methods

### 2.1. Reagents

Polydatin (PD; with a purity ≥98%, HPLC; molecular weight: 390.39) was purchased from Beijing Solarbio Science & Technology Co., Ltd. (Cat # 27208-80-6; Beijing, China). Dulbecco's Modified Eagle's Medium (DMEM), fetal bovine serum (FBS), and 0.25% Trypsin—EDTA were purchased from Gibco Life Technologies (MD, USA). Insulin, dexamethasone, 3-isobutyl-1-methylxanthine (IBMX), and Oil red O were obtained from Sigma-Aldrich (MO, USA). Primers were obtained from Sangon Biotech Company (Shanghai, China). Mouse insulin ELISA kit was obtained from Mercodia (Cat # 10-1247-01; Uppsala, Sweden). The commercial kits for triglyceride (TG) was purchased from Abbott Molecular (IL, USA), and low-density lipoprotein (LDL), and high-density lipoprotein (HDL) were obtained from Biosino Biotechnology Co., Ltd. (Beijing, China). Anti-MCP1 was obtained from Abcam Trading Company Ltd. (Cat # ab25124; Cambridge, UK), anti-PPAR*γ* was from Santa Cruz Biotechnology (Cat # sc-7273; CA, USA), anti-Leptin was from Proteintech Group (Cat # 17436-1-AP; IL, USA), anti-*β*-actin was from Earth Ox (Cat #E021020-01; CA, USA), and anti-TNF-*α* (Cat # 11948) and anti-GAPDH (Cat # 97166) were from Cell Signaling Technology (MA, USA). PrimeScript™ RT Master Mix for reverse transcription was obtained from Takara Bio Inc. (Kyoto, Japan). SYBR Green PCR Master Mix was obtained from ABI Life Tech. (CA, USA).

### 2.2. 3T3-L1 Cell Culture and Treatment

Mouse 3T3-L1 preadipocytes were purchased from Chinese Academy of Sciences Cell Bank (Shanghai, China). They were cultured in DMEM supplemented with 10% FBS and antibiotics (1% penicillin/streptomycin). 5 × 10^5^ cells were seeded in each well of six-well plate. Adipocyte differentiation was conducted following the previously published protocols [21], with few modifications. 2 days after 100% confluence (Day 0), the cells were treated with a differentiation medium consisting of 10 mg/L insulin, 0.5 mM IBMX, 1 *μ*M dexamethasone, and with or without 20 *μ*M PD in DMEM + 10% FBS for 2 days (Day 2). Then the medium was replaced with DMEM + 10% FBS supplemented with 10 mg/L insulin for 2 days (Day 4). Thereafter, the medium was changed every 2 days with DMEM + 10% FBS until the cells were fully differentiated (Day 8).

### 2.3. Oil Red O Staining and Quantitation

On day 8, the cells were washed three times with phosphate-buffered saline (PBS) and then fixed for 30 min with 4% buffered paraformaldehyde. Then, cells were stained with Oil red O for 20 min following the manufacture's instruction. After washed three times with PBS, the lipid droplets were observed and photographed under a microscope (Axio Observer A1; Carl Zeiss, Göttingen, Germany). To measure the total content of lipids in each well, Oil-Red-O-stained lipids were dissolved in 100% isopropanol, and the absorbance of each sample was measured at 490 nm using a Multiskan GO microplate reader (Thermo Fisher, MA, USA).

### 2.4. Animal Model and PD Treatment

Thirty healthy specific pathogen free C57BL/6J male mice (abbreviated by C57) aged 5 weeks were supplied by Shanghai Sippr-BK Laboratory Animal Co., Ltd. (Shanghai, China; animal quality certification number: SCXK 2013-0016). The mice were adapted to the environment for 1 week and then randomly divided into 3 groups based on the weight as follows: (1) normal group (*n* = 10) fed with standard chow diet (10% kcal fat, 70% kcal carbohydrates, 20% kcal proteins) for 14 weeks; (2) HFD group (*n* = 10) fed with high-fat diet (45% kcal fat, 35% kcal carbohydrates, 20% kcal protein; Medicience Ltd., Yangzhou, China) for 14 weeks; (3) PD treatment group (*n* = 10) fed with HFD for 10 weeks and then switched to HFD supplemented with PD (100 mg/kg/day, suspended in 0.5% CMC-Na solution) by oral gavage for another 4 weeks. Animals were housed under barrier system at the Experimental Animal Center of Zhejiang Academy of Medical Sciences, with a temperature (20–25°C) and humidity (40–70%) with 12:12 hour light and dark cycle. All experiments were conducted according to the institutional guidelines for animal care and the experimental procedures were approved by the Animal Research Ethics Committee of the Second Affiliated Hospital of Jiaxing University (Jiaxing, China).

### 2.5. Plasma and Adipose Tissue Collection

At the end of experiment, all animals were fasted for 12 h and blood samples were collected from retinal venous plexus. Serum was harvested by centrifuging (2500*g*; 10 min) and stored at −80°C for ELISA and metabolic parameters analysis. The retroperitoneal and epididymal adipose tissues were isolated and frozen in liquid nitrogen, and fixed in 10% neutral buffered formalin, respectively.

### 2.6. General Biochemical Parameters in Blood

The levels of TG, HDL, LDL, and blood glucose in serum were determined using the Abbott Architect C16000 automatic biochemical analyzer (Abbott Diagnostics, Germany). The insulin concentrations were measured by ELISA according to the manufacturer's instructions.

### 2.7. Histology Analysis

#### 2.7.1. Hematoxylin-Eosin (H&E) Staining

Adipose tissues were fixed in 4% buffered paraformaldehyde, embedded in paraffin, and sectioned at 4 *μ*m thickness. The histological characterizations, including adipocyte sizes, were observes by hematoxylin-eosin (H&E) staining. Five random fields from each section were examined, and adipocyte diameter was measured using AxioVision Rel. 4.6.3 (Carl Zeiss, Göttingen, Germany).

#### 2.7.2. Immunohistochemistry (IHC)

Paraffin-embedded fat tissues were sliced into 4 *μ*m thick tissue sections, followed by being deparaffinized and incubated with anti-Leptin antibody (Proteintech, IL, USA) at a dilution of 1 : 500 at 4°C overnight. Slides were then incubated with biotinylated anti-rabbit IgG (Servicebio, Hubei, China) for 30 min at room temperature. After DAB solution staining, slides were counterstained with hematoxylin, dehydrated, and mounted. The slides were observed and photographed using AxioVision Rel. 4.6.3 (Carl Zeiss, Göttingen, Germany).

### 2.8. RNA Extraction and Real-Time PCR Analysis

Total RNA was extracted from 3T3-L1 mature adipocytes and mice adipose tissue using Trizol reagent (Invitrogen, CA, USA) and reversely transcribed into cDNA using PrimeScript™ RT Master Mix (Takara, Kyoto, Japan). The resulting cDNA were amplified using SYBR Green PCR Master Mix (ABI, CA, USA). Quantitative polymerase chain reaction (qPCR) was performed on an ABI StepOne plus real-time PCR system (ABI, CA, USA). The primers used are listed in Table 1. Samples were assayed in triplicate, and the relative abundance of mRNAs was normalized to GAPDH gene expression using 2^−∆∆Ct^ method.

### 2.9. Western Blot

Proteins were extracted from mice adipose tissue in 1 × SDS lysis buffer (150 mM NaCl, 25 mM Tris-HCl, pH 7.6, 1% sodium deoxycholate, and 1% NP-40) with a protease inhibitor cocktail (Roche, Switzerland). Protein expression in mice retroperitoneal and epididymal adipose tissues were measured by Western blot with antibodies obtained from Abcam (MCP-1; 1 : 300), Santa Cruz Biotechnology (PPAR*γ*; 1 : 2000), Earth Ox (*β*-actin; 1 : 1000), and Cell Signaling Technology (TNF-*α*; 1 : 500 & GAPDH; 1 : 600). Protein was separated by SDS-PAGE and transferred to polyvinylidene difluoride (PVDF) membranes (Millipore, MA, USA). The membranes were blocked with 5% fat-free milk and incubated with different primary antibodies at 4°C. The bound antibodies were detected using horseradish peroxidase-conjugated anti-rabbit antibodies or anti-mouse antibodies (Jackson Laboratory, ME, USA; 1 : 5,000) for 1.5 h at room temperature. Protein levels were detected with SuperSignal West Pico ECL buffer (Thermo Fisher, IL, USA) using ChemiDoc XRS imaging system (BioRad, CA, USA). The intensity of protein bands was quantitated using Image J analysis software (Version 1.50i; MD, USA).

### 2.10. Statistical Analysis

Statistical analyses were performed using GraphPad Prism 6.0 (GraphPad Software, CA, USA). Comparisons between two groups were performed by the two-tailed unpaired *t* test. Data are presented as mean ± SD. Differences were considered significant at *P* < 0.05.

## 3. Results

### 3.1. PD Inhibited the Adipogenesis of 3T3-L1 by Downregulating the Expression of PPAR*γ*

**Figure 1:**
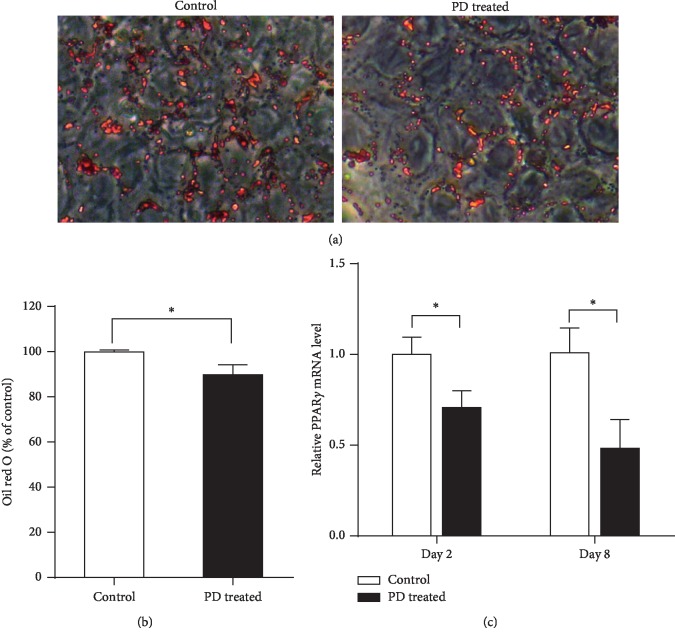
Effects of PD on lipid accumulation. 3T3-L1 preadipocytes were treated with 20 *μ*M PD during differentiation. (a) Oil Red O staining was performed on day 8 after induction of differentiation. (b) 3T3-L1 adipocytes positive for Oil Red O staining were quantified. (c) Effect of PD on PPAR*γ* mRNA expression levels of 3T3-L1 adipocytes on day 2 and day 8. All results are expressed as mean ± SD. ^*∗*^*P* < 0.05.

To confirm the effects of PD on the differentiation of 3T3-L1 based on our previous study [20], lipid accumulation in 3T3-L1 adipocytes were investigated by Oil Red O staining. 3T3-L1 preadipocytes were induced differentiation into mature adipocytes and treated with 0 or 20 *μ*M PD for 2 days. As expected, cells treated with PD had less lipid droplets compared with the control group on day 8 (Figure 1(a)). Quantification of Oil Red O staining demonstrated that PD treatment significantly decreased lipid accumulation compared with the control group (*P* < 0.05; Figure 1(b)), indicating that PD inhibited the adipogenesis of 3T3-L1. Furthermore, we examined the mRNA expression of PPAR*γ* on day 2 and day 8. The results showed that the mRNA expression level of PPAR*γ* was lower in the PD treated group compared with the control group (*P* < 0.05; Figure 1(c)).

### 3.2. Effects of PD on Body Weights, Adipose Tissue Mass, and Biochemical Parameters in HFD-Fed Mice

**Figure 2:**
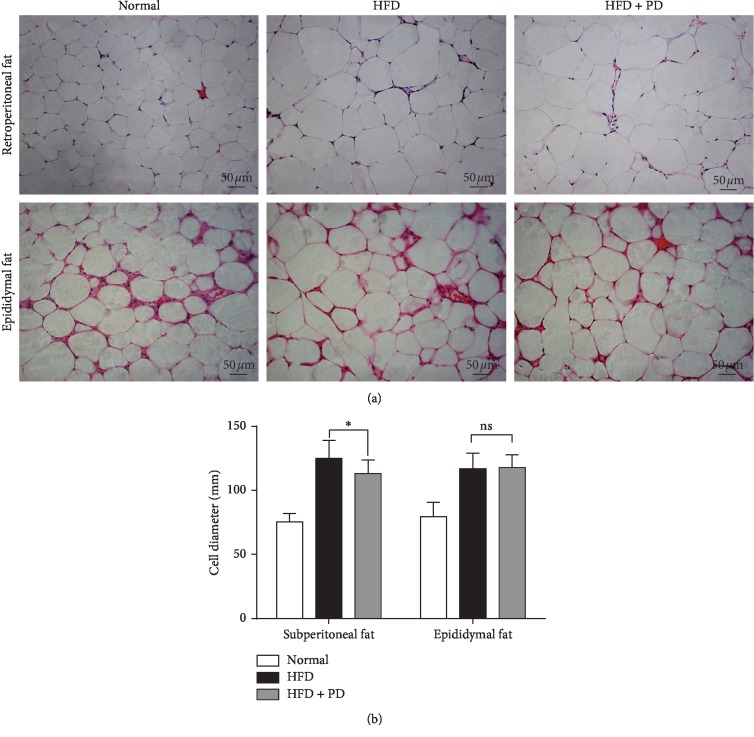
Effects of PD on retroperitoneal and epididymal adipocyte sizes in HFD-fed mice by hematoxylin-eosin staining (200x). Normal, normal group; HFD, HFD-fed group; HFD + PD group, HFD supplemented with 100 mg/kg/day PD group. All results are expressed as mean ± SD. ns., no significant differences; ^*∗*^*P* < 0.05.

In this study, 100 mg/kg/day PD was used following the previously published dose with a better effect [22]. The average body weight was significantly lower in the HFD + PD group compared with the HFD group (39.44 ± 0.61 g vs. 41.50 ± 0.50 g, *P* < 0.05; Table 2), as well as retroperitoneal adipose tissue weight (0.89 ± 0.05 g vs. 1.12 ± 0.05 g, *P* < 0.05; Table 2), and this decrease was accompanied by a significant decrease in retroperitoneal adipocyte sizes in the HFD + PD group compared with the HFD group (113.0 ± 1.5 vs. 125.2 ± 2.0 *μ*m, *P* < 0.05; Figure 2(b)), but not epididymal adipocyte sizes (118.0 ± 1.4 vs. 116.9 ± 1.7 *μ*m; Figure 2(b)). In order to detect the effects of PD on lipid and glucose metabolism, we tested the concentration of glucose, TG, HDL, LDL, and insulin in the serum of HFD-fed mice. The results demonstrated that PD decreased TG and LDL levels (*P* < 0.05, Table 2). Moreover, PD significantly increased HDL concentration in the serum of HFD-fed mice (*P* < 0.05; Table 2). However, we did not observe changes of serum glucose and insulin concentration after PD treatment (Table 2).

### 3.3. The mRNA Expression of PPAR*γ* and C/EBP*α* in the Retroperitoneal and Epididymal of HFD-Fed Mice

**Figure 3:**
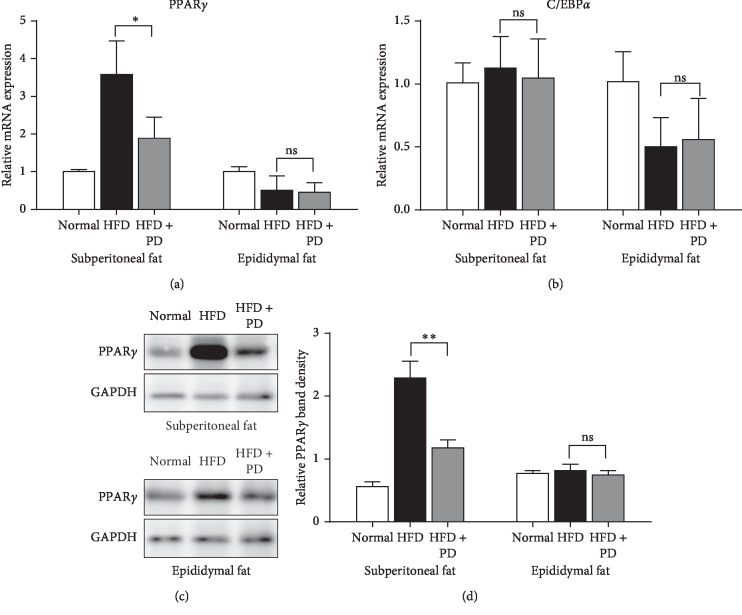
Effects of PD on differentiation makers, PPAR*γ* and C/EBP*α*, in HFD-fed mice. (a) PPAR*γ* mRNA expression levels of adipose tissues after treatment of PD in HFD-fed mice. (b) C/EBP*α* mRNA expression levels in HFD-fed mice. (c) Representative images for western blot of PPAR*γ* in HFD-fed mice. (d) Quantitative analyses of the immunoblots were shown for PPAR*γ* in HFD mice. Normal, normal group; HFD, HFD-fed group; HFD + PD group, HFD supplemented with 100 mg/kg/day PD group. All results were expressed as mean ± SD. ns., no significant differences; ^*∗*^*P* < 0.05; ^*∗∗*^*P* < 0.01.

In the HFD-fed mice, PD downregulated the mRNA and protein expressions of PPAR*γ* in the retroperitoneal fat, but not in epididymal fat (Figures 3(a), 3(c), and 3(d)). However, no significant changes were observed for C/EBP*α* mRNA expressions both in retroperitoneal and epididymal fat (Figure 3(b)).

### 3.4. PD Upregulated the Expression of Leptin in the Adipose Tissues of HFD-Fed Mice

**Figure 4:**
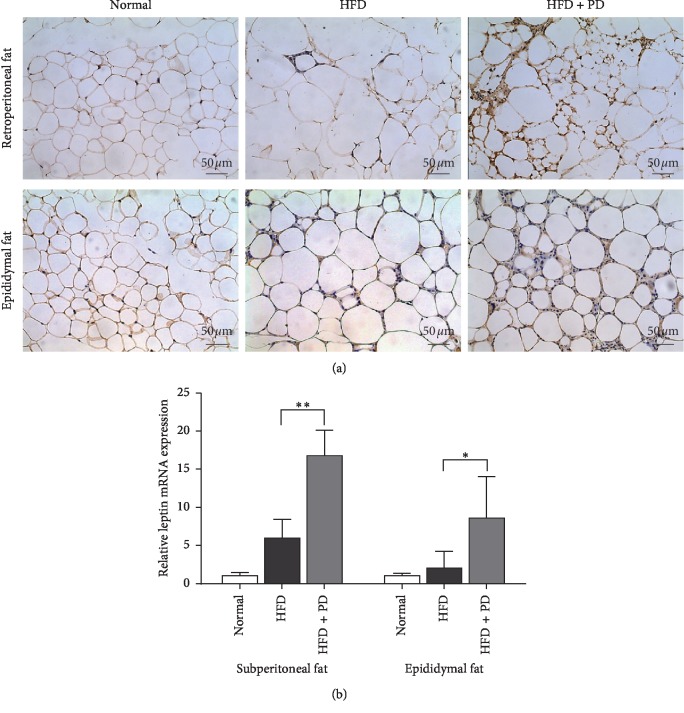
Effects of PD on leptin expressions in HFD-fed mice. (a) Effects of PD on leptin expression in retroperitoneal and epididymal adipose tissues of HFD-fed mice. (b) Leptin mRNA expression levels of adipose tissues after treatment of PD in HFD-fed mice. Normal, normal group; HFD, HFD-fed group; HFD + PD group, HFD supplemented with 100 mg/kg/day PD group. All results were expressed as mean ± SD. ^*∗*^*P* < 0.05; ^*∗∗*^*P* < 0.01.

In the HFD-induced mice, compared with HFD group, PD increased the mRNA expressions of leptin in the retroperitoneal fat (*P* < 0.01) and epididymal fat tissues (*P* < 0.05; Figure 4(b)) in the HFD + PD group with a mean fold change of 2.8 and 2.2 (relative to HFD group), respectively. Immunohistochemistry staining showed that PD improved leptin expression both in retroperitoneal and epididymal fat tissues of HFD-fed mice (Figure 4(a)), which was consistent with the mRNA expressions.

### 3.5. PD Decreased the Inflammatory State of Adipose Tissue in HFD-Fed Mice

**Figure 5:**
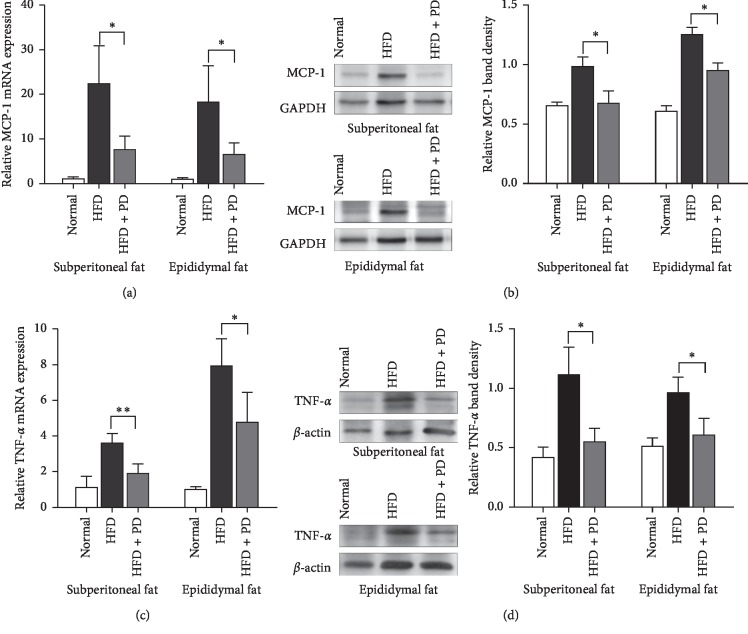
Effects of PD on inflammation-related genes, MCP-1 and TNF-*α*, in HFD-fed mice. (a) MCP-1 mRNA expression levels of retroperitoneal and epididymal adipose tissues after treatment of PD in HFD-fed mice. (b) Representative images and quantitative analyses for western blot of MCP-1 in HFD-fed mice. (c) TNF-*α* mRNA expression levels in HFD-fed mice. (d) Representative images and quantitative analyses for western blot of TNF-*α* in HFD-fed mice. Normal, normal group; HFD, HFD-fed group; HFD + PD group, HFD supplemented with 100 mg/kg/day PD group. All results were expressed as mean ± SD. ^*∗*^*P* < 0.05; ^*∗∗*^*P* < 0.01.

In parallel, the mRNA expression of proinflammatory related genes were measured in the adipose tissues of HFD-fed mice. Compared to these in HFD group, oral treatment of mice with PD significantly reduced mRNA expression of the following proinflammatory cytokines, MCP-1 and TNF-*α*, in the retroperitoneal and epididymal fat tissues (*P* < 0.05; Figures 5(a) and 5(c)). Simultaneously, PD decreased the protein expression of MCP-1 and TNF-*α* in both fat tissues of HFD-fed mice (Figures 5(b) and 5(d)).

## 4. Discussion

Currently, the effects of PD on the regulation of glucose and lipid metabolism, adipogenesis, and inflammation in obesity have not fully understood. In this study, we provide evidence that PD can ameliorate lipid metabolism and decrease retroperitoneal fat weight and body weight, at least partially by the inhibition of adipogenesis in the retroperitoneal adipose tissue in HFD-fed model. Moreover, PD presented anti-inflammatory action in HFD-fed mice model.

Glucose and lipid metabolism disorders are the main characteristics of obesity [1]. In order to verify the effects of PD on glucose and lipid metabolism, we testified it in the HFD-fed mice model. In our study, although no obvious changes in glucose was observed, dietary PD supplement prevented lipid metabolic disorder in HFD mice. In the HFD-fed mice, PD decreased retroperitoneal adipose cell sizes, retroperitoneal fat weight, and body weight; significantly lowered serum TG and LDL levels; and simultaneously increased HDL levels compared with the HFD control mice (Table 2; Figure 2). A study using a leptin receptor-deficient *db/db* mice model reported that PD could regulate lipid metabolism by decreasing TC, TG, and LDL levels [23]. Our results were consistent with the previous study, which suggested the effects of PD on lipid metabolism regulation. Together with our *in vitro* study (Figure 1), using 3T3-L1 preadipocytes, indicated that PD could ameliorate lipid metabolism at least partially by inhibiting adipogenesis and lipid accumulation during the preadipocytes differentiation. Thus, PD may serve as an activity component that can ameliorate lipid metabolism in HFD-induced obesity.

Subsequently, in order to study the regulation effects of PD on adipogenesis, we detected the expression levels of PPAR*γ* and C/EBP*α* in the adipose tissues of HFD-fed mice after PD treatment (Figure 3). The results showed that PD downregulated PPAR*γ* mRNA and protein expressions in the retroperitoneal fat tissues of HFD-fed mice. The results were consistent with our *in vitro* study that PD inhibited the differentiation of 3T3-L1 preadipocytes into mature adipocytes by suppressing the expression of PPAR*γ* (Figure 3(c)), which is a key transcriptional regulator of adipocyte differentiation [6, 24, 25]. However, we did not observe the same results in the epididymal fat tissue of HFD-fed mice. These differences are considered to be involved in the divergence effects of PD on different adipose tissues. White adipose tissue (WAT) is distributed throughout the body surrounding visceral organs, in subcutaneous regions, and in the face [4]. Subcutaneous and visceral WAT are thought to have distinct tissue-specific metabolic functions, and particularly accumulation of visceral fat during the development of obesity is correlated with chronic systematic inflammation and insulin resistance [26]. Our results indicated that PD differentially affected the characteristics of retroperitoneal WAT and epididymal WAT depots in obese mice, even though both of them belonged to visceral WAT. Meanwhile, we detected the mRNA expressions of C/EBP*α*, which functions as a principal player in adipogenesis [5], in the adipose tissue of HFD-fed mice model. Unfortunately, no obvious changes of C/EBP*α* mRNA expression levels were observed after PD treatment in the HFD-fed mice. C/EBP*α* is expressed at the adipogenic initiation stage and synergistically triggers adipocyte-specific gene expression with PPAR*γ* after the growth arrest stage [27]. In our previous *in vitro* study, low concentration of PD inhibited the mRNA expression of C/EBP*α* on day 2 during the differentiation of 3T3-L1 preadipocytes, indicating that PD might regulate the expression of C/EBP*α* at the adipogenic initiation stage. Thus, the combined results from our *in vivo* and *in vitro* experiments suggest that PPAR*γ* might be a target of PD to affect adipogenesis and lipid metabolism in HFD-induced obesity.

In addition, we detected that PD increased the leptin mRNA and protein expression levels in the retroperitoneal and epididymal adipose tissues of HFD-fed mice (Figure 4). Leptin is an adipocyte-derived hormone primarily synthesized and secreted by the white adipose tissue [28, 29], which acts through the hypothalamus and other brain regions, including the reward system [30]. Leptin is involved in food intake and energy balance, and genetic mutations in the leptin pathway can cause obesity in humans and rodents [30, 31]. For example, leptin-deficient *ob/ob* mice are one of the most investigated mice models of obesity that represent morbid obesity, insulin resistance, hyperphagia, and infertility [30]. In patients with genetic deficiency of leptin, exogenous leptin therapy effectively controls hyperphagia and corrects metabolic disorders [32]. In our previous study, PD upregulated the mRNA level of leptin in 3T3-L1 cells [20]. Altogether, considering the reduction effect of PD in adipose tissue mass and body weight in HFD-fed mice, our results indicate that PD is involved in regulation of leptin expression in obesity and thus might affect body weight. However, the effects of PD on leptin expression and its underlying mechanisms are needed to be clarified in the future.

Adipose tissue inflammation is a major cause of obesity-associated syndromes [8, 9] and therefore may serve as potential targets for supporting therapies chronic inflammation. Under an altered homeostatic condition, obesity presents certain features of a continuing low-grade systemic chronic inflammation, including elevated levels of inflammatory adipokines. Numerous pharmacologic investigations have reported the anti-inflammatory effects of PD by modulating the expression of inflammatory cytokines [17], including TNF-a, IL-6, IL-1*β*, and others. PD decreases the expression of TNF-a and IL-1*β* at mRNA and protein levels through downregulating NFkB p65 activity and expression [33, 34]. Our results demonstrated that PD could downregulate the expression levels of proinflammatory cytokines, MCP-1 and TNF-*α*, in the adipose tissues of HFD-fed mice (Figure 5). MCP-1 plays a key role in attracting macrophage infiltration into adipose tissue, resulting in increased adipose tissue inflammation in obesity [35, 36]. TNF-*α* is a key secretory cytokine in adipocyte tissue, whose role in obesity has been linked to proinflammation and insulin resistance [10, 37, 38]. Adipose tissue is described as an important endocrine organ, with the ability to modulate lipid metabolism and peripheral inflammation [39]. Our inference was verified both *in vitro* and *in vivo* experiments. Consistent with our previous study, which showed that the mRNA levels of MCP-1 and TNF-*α* were downregulated by administration of PD using 3T3-L1 cells, our results suggests that PD is involved in regulation of inflammatory state of adipose tissue and thus possibly relieve the systematic chronic inflammation in obesity.

## 5. Conclusions

In conclusion, our data demonstrate that oral administration of PD could be of relevance for its potential therapeutic effects in obesity-related lipid metabolism, anti-inflammation, and body weight loss. Thus, PD may have potential for a future pharmacological drug in the treatment of obesity and its associated metabolic diseases.

## Figures and Tables

**Table 1:** Primers for qRT-PCR amplification of indicated genes.

Gene	Forward primer (5′-3′)	Reverse primer (5′-3′)
GAPDH	AACAGGGTGGTGGACCTCAT	GGGATAGGGCCTCTCTTGCT
MCP-1	CACAACCACCTCAAGCAC	AAGGGAATACCATAACATCA
TNF-*α*	AGGGAGAGTGGTCAGGTTGC	GTGAGGAAGGCTGTGCATTG
PPAR*γ*	TCGCTGATGCACTGCCTATG	GAGAGGTCCACAGAGCTGATT
C/EBP*α*	CAAGAACAGCAACGAGTACCG	GTCACTGGTCAACTCCAGCAC
Leptin	CCAGGATCAATGACATTTCACACAC	AGGTCATTGGCTATCTGCAGCAC

**Table 2:** Effects of PD on general characteristics of HFD-fed mice.

Parameter	Normal (*n* = 10)	HFD (*n* = 10)	HFD + PD (*n* = 10)
Body weight (g)	29.40 ± 0.44	41.50 ± 0.50^###^	39.44 ± 0.61^*∗*^
Retroperitoneal fat (g)	0.37 ± 0.03	1.12 ± 0.05^###^	0.89 ± 0.05^*∗*^
Epididymal fat (g)	0.78 ± 0.05	1.07 ± 0.04^###^	1.05 ± 0.03
Glucose (mmol/L)	3.10 ± 0.18	5.69 ± 0.23^##^	5.68 ± 0.19
TG (mmol/L)	0.88 ± 0.05	1.36 ± 0.04^##^	1.07 ± 0.08^*∗*^
HDL (mmol/L)	1.81 ± 0.03	1.91 ± 0.07	2.19 ± 0.09^*∗*^
LDL (mmol/L)	0.37 ± 0.01	0.94 ± 0.04^###^	0.79 ± 0.06^*∗*^
Insulin (*μ*g/L)	0.93 ± 0.22	4.11 ± 0.70^###^	3.60 ± 0.78

Normal, normal group; HFD, HFD-fed group; HFD + PD group, HFD supplemented with 100 mg/kg/day PD group. All results are expressed as mean ± SD. ^##^*P* < 0.01, ^###^*P* < 0.001, compared with the normal group; ^*∗*^*P* < 0.05, compared with the HFD group.

## Data Availability

The data used to support the findings of this study are available from the corresponding author upon request.
